# On Intemperance, as a Disease, and as Susceptible of Cure

**Published:** 1829-01-01

**Authors:** 


### On Intemperance, considered as a Disease, and as susceptible of Cure. By J. H. Kain, M.D.

One of our transantlantic brethren has chosen the above subject for an essay in the American Journal of the Medical Sciences; and has really made many interesting observations on that melancholy addiction to intoxication which prevails more or less in all countries—but very widely in the United States. Dr. K. if he does not advocate the cause of the drunkard, has laboured hard to palliate the offence of drunkenness—to remove it, in fact, from the class of *moral vices*, and place it as one of our *physical maladies*. Bulimia, salacitas, and nymphomania, (he observes) diseases in which natural appetites and passions are morbidly excited, have found their places in all systems of medical nosology—whereas, a morbid thirst for intoxicating liquors has only been considered as a *moral disease*, and, for its cure, *moral* remedies have been thought sufficient. As well (he thinks) might we address arguments to a burning fever, as to expect, by moral means, to appease the anorexia and gnawing sensations, which, in the drunkard, solicit so importunately for the inebriating draught! Here, it is evident that the ingenious author confounds the physical *effects* of habitual inebriety, with the *moral laxity* of principle that led to the confirmation of the habit. As well might he identify a chancre or bubo with the dissolute life that preceded it—debt with gambling—or a broken head with a passion for skating. The author observes that, "men whose conduct, in their lucid moments, has been most unexceptionable—whose moral worth cannot be disputed—and whose talents are often enviable—such men, in consequence of an unhappy *propensity*, (which I hope to prove a disease) have been branded with infamy—and even physicians have united with the pulpit and the press, to reduce them to a level with brutes." We admit that this *propensity*, once established, is a *disease*, and as such, that it comes more under the medicinal treatment of the physician than the declamation of the moralist or divine; but still we cannot shut our eyes to the fact, that this *propensity* is not originally caused by "anorexia, or gnawing sensations" in the stomach, but by the indulgence of the passions—the love of mirth and carousing—and the temporary pleasure of vinous and conversational excitement. Let us hear the author himself.

"After an *evening debauch*, or what the patient would call a party of pleasure, he feels a want of appetite when he comes to his breakfast the next morning: this is readily relieved by a glass of bitters or a cup of very strong coffee, and the enfeebled stomach is goaded on to perform wonders in the eating way. The consequence of such exertion is an impaired digestion, and a heavy, horrible sensation is felt in the region of the stomach. To this is added an indescribably gloomy state of mind. The patient finds business burdensome and solitude insupportable. He flies to the tavern or the doctor, to his medicine chest or the brandy bottle, and soon obtains a temporary truce. A glass of toddy, punch, or sling, soothes his outraged stomach, and prepares him for dinner. If he have no dinner, another glass or two suits him as well, and what he fails in eating he makes up in drinking. It is unnecessary to pursue the history further. He rises the next morning with increased anorexia, nausea, or vomiting. This is alarming, but is often immediately relieved with warm spiced toddy, peppermint, or capsicum and whiskey, and he is prepared to pass the day as he did the last."

Now, if the individual will embrace the evening debauch or the party of pleasure, *after* clearly experiencing the *effects* of the indulgence, we must and ought to censure his want of moral firmness or resolution to resist the temptation. We agree with the author, that the propensity once established, "its seat is in the stomach, and is no doubt produced by perverted or disordered action of that viscus." But wherever the *seat* may be, the whole man, intellectual and physical, feels the dire effects, and the wretched sensations can only be lulled by re-applications of the poison that produced them. To him, the bottle is a catholicon. It not only relieves gastrodynia, nausea, and colic, but the gloomy emotions of the mind, which are worse than all. It transforms this thorny, rugged wilderness of a world into a Paradise! How can we wonder that, under such circumstances, moral reasonings are vain! The sufferings of the drunkard, while sober, are beyond the imagination of the temperate—and the feelings of pleasure which he derives from the bottle are, no doubt, exaggerated, by the sudden release from the tortures of the damned. But to come to the remedy—if remedy there be, for this *acquired*, not inflicted, disease.

It is to be borne in mind, that when drunkenness has become a disease, viz. when the *habit* is completely formed, the incitement to inebriation is two-fold—the horrors of the sober state, and the pleasures of the intoxicated condition. Although a man may be firmly convinced that the misery of the one state is the inevitable consequence of the unnatural excitation of the other, he will *endure* the one for the sake of enjoying the other! Hence it is, that arguments are generally useless; and to break the habit of inebriety, we must contrive to diminish, annihilate, or reverse the pleasurable sensations attendant on it. To effect this purpose, we may profitably employ the knowledge of a law in the animal economy, which is but little studied or understood at present. The stomach is possessed of two kinds of sensibility—common and organic. The latter may be pleased or offended without the consciousness of the individual. Thus, certain kinds of food will disagree with the stomach, (as far as its organic sensibilities are concerned) and induce such an *antipathy* to that particular article of nutriment, that nothing can induce the individual to taste it—and all this without the individual knowing why. In some parts of Scotland and Ireland this antipathy is known among the vulgar by the term "scunder," or sickly aversion to any particular species of aliment. Milk, for instance, will disagree with a stomach, and that without any pain, nausea, or vomiting, but merely by offending the organic sensibilities of the viscus—and from that moment the very sight of milk will induce the most distressing sensations of disgust. It is the same with medicines. We have known digitalis produce the "Scunder"—and that to such a degree, that the patient would rather die than take the medicine. The same we have seen from ipecacuanha, antimony, and some other medicaments. Here, then, is the key to the anti-inebriating specifics—and especially to one which has excited considerable attention in America—namely, Chambers' nostrum. There is not the smallest doubt that it is principally composed of tartrite of antimony. We have known several instances, where drunken stewards of mess-rooms, and tipling butlers, have been cured—at least for a time, of their intemperate habits, by the admixture of tartar emetic with the wine or spirits that were purposely left in their way. We have known more than one instance, in high life, where the "Scunder" was effectually induced by a few grains of antimony. One of these cases we published some years ago. It appears that Dr. Rush cured a negro of inebriety, by infusing tartar-emetic in his grog, and Dr. Kain avers that he has effectually secured numbers against this dreadful propensity. It need hardly be remarked, that the antimony must be given by stealth, and in the drunkard's favourite beverage, otherwise the charm will be ineffectual, and the antipathy will not be engendered against the inebriating material. On this account, the remedy can only be applied by the friends of the intemperate, under the direction of the medical practitioner. If the antipathy can once be established, it will be in vain for the individual to repair to the tavern. The purest liquor will be equally detested as that which was originally adulterated with the antimony. Possessing no positive taste itself, the tartrite of antimony communicates a disgusting quality to every fluid in which it is dissolved. "I have often seen," says Dr. Kain, "persons who, from taking our medicine in the form of vinum antimonii, could never afterwards drink wine."

"I may be permitted here to remark, that while I claim no credit for originality in the application of our remedy, I had used it in this disease for some time before I saw the analysis of Chambers's remedy for intemperance by the Committee of the New York Medical Society. My method of prescribing it has varied according to the habits, age, and constitution of the patient. I give it only in alterative and slightly nauseating doses. A convenient preparation of the medicine is 8 grs. of tartrite of antimony dissolved in ℥iv. of boiling water—℥ss. of the solution to be put into a half pint, pint, or quart of the patient's favourite liquor, and to be taken daily in divided portions. If severe vomiting or purging ensue, I should direct laudanum, to allay the irritation, and diminish the dose. In every patient it should be varied according to its effects. In one instance, in a patient who lived ten miles from me, severe vomiting was produced, more, I think, from excessive drinking than the use of the remedy. He recovered from it, however, without any other bad effects. In some cases the change suddenly produced in the patient's habits has produced considerable lassitude and debility, which was of but short duration. In a majority of cases no other effect has been perceptible than slight nausea, some diarrhœa, and a gradual but very uniform distaste to the menstruum."

Dr. Kain cannot vouch for the permanency of the cure in this way. A temporary respite, however, is pretty certain —and this, in many cases, would be an important advantage, as affording a favourable opportunity for moral arguments, and that at a time when the inebriating potation excited disgust instead of pleasure. It is possible that mischief might sometimes result from the incautious use, or rather abuse of the remedy. Drunkards, more than any other people, are subject to chronic inflammations of the viscera; and, under such circumstances, large doses of tartrite of antimony might do harm. It should not, however, be employed in doses to excite vomiting. It more effectually produces the ANTIPATHY in smaller doses longer continued, by which the organic, rather than the common sensibility of the stomach is offended.

